# Antitumor Effects of Recombinant Antivascular Protein ABRaA-VEGF_121_ Combined with IL-12 Gene Therapy

**DOI:** 10.1007/s00005-013-0259-5

**Published:** 2013-11-13

**Authors:** Agnieszka Ciomber, Andrzej Smagur, Iwona Mitrus, Tomasz Cichoń, Ryszard Smolarczyk, Aleksander Sochanik, Stanisław Szala, Magdalena Jarosz

**Affiliations:** 1 Center for Translational Research and Molecular Biology of Cancer, Maria Skłodowska-Curie Memorial Cancer Center and Institute of Oncology, Gliwice Branch, Wybrzeze Armii Krajowej 15, 44-101 Gliwice, Poland; 2 Department of Bone Marrow Transplantation, Maria Skłodowska-Curie Memorial Cancer Center and Institute of Oncology, Gliwice Branch, Gliwice, Poland

**Keywords:** Combined therapy, Antivascular strategy, ABRaA**-**VEGF_121_ protein, Immunostimulation, IL-12

## Abstract

Development and neoplastic progression strongly rely on tumor microenvironment cells. Various kinds of cells that form such tumor milieu play substantial roles in angiogenesis and immunosuppression. Attempts to inhibit tumor vascularization alter tumor milieu and enhance immune response against the tumor. Anticancer therapeutic strategy bringing together antiangiogenic and immunostimulating agents has emerged as a promising approach. We here investigated whether therapy directed against preexisting vessels, combined with an immunomodulatory factor would be equally effective in arresting tumor growth. To this goal, we investigated the effectiveness of ABRaA**-**vascular endothelial growth factor isoform 121 (VEGF_121_), an antivascular drug constructed by us. It is a fusion protein composed of VEGF_121_, and abrin A chain (translation-inhibiting toxin). We used it in combination with interleukin (IL-12) gene therapy and tried to inhibit B16-F10 melanoma tumor growth. ABRaA**-**VEGF_121_ is a chimeric recombinant protein capable of destroying tumor vasculature and triggering necrosis in the vicinity of damaged vessels. IL-12 cytokine, in turn, activates both specific and non-specific immune responses. Our results demonstrate that combination of ABRaA**-**VEGF_121_ antivascular agent with immunostimulatory cytokine IL-12 indeed inhibits tumor growth more effectively than either agent alone, leading to complete cure of ca. 20 % mice. Post-therapeutic analysis of tumors excised from mice treated with combination therapy showed decreased numbers of blood microvessels in the tumor microenvironment, lowered numbers of regulatory T lymphocytes, as well as showed higher levels of CD4^+^ and CD8^+^ as compared to control mice. It seems that bringing together antivascular strategy and the action of immunostimulating agents indeed inhibits growth of tumors.

## Introduction

Tumor progression is strongly dependent on its microenvironment (Szala et al. 2010). Various kinds of cells (T lymphocytes, dendritic cells, natural killer cells, macrophages and neutrophils) that form tumor milieu play significant roles in processes crucial for unrestrained tumor development, i.e., angiogenesis and immunosuppression (Shurin et al. 2012). Generally, cells forming tumor milieu create a specific microenvironment which is selective towards cancer cells with proangiogenic and immunosuppressive phenotype (Hofmeister et al. 2008; Mocellin and Nitti 2008; Szala et al. 2010). A specific role is played here by vascular endothelial growth factor (VEGF) which acts both in proangiogenic and immunosuppressive manner. VEGF is the main proangiogenic agent released by cancer cells as well as the main immunosuppressant inhibiting maturation of dendritic cells (Balkwill 2009; Tartour et al. 2011). However, prolonged antiangiogenic therapy may result in drug resistance of cancer cells. They cease to react to therapeutic agents and may become more invasive (Ellis and Hicklin 2008). Thus, antiangiogenic strategy alone is not a satisfactory therapeutic approach (Azam et al. 2010). In order to render anticancer strategy more effectively, therapy should be directed not only against cancer cells; instead, it should target whole tumor microenvironment (Jinushi and Dranoff 2007; Noonan et al. 2008; Szala et al. 2010). In this respect, a promising therapeutic approach emerges by bringing together antiangiogenic and immunostimulating agents (Terme et al. 2012). Such a combination should also eliminate cancer stem cells (Szala et al. 2010).

In our investigation, we examined therapeutic effects of an experimental approach directed against preexisting vessels combined with immunomodulatory factor. In our study, we tested a combination involving ABRaA**-**VEGF_121_, an antivascular fusion protein, and IL-12, an immunostimulating cytokine. ABRaA**-**VEGF_121_ is a fusion recombinant protein composed of VEGF isoform 121 (VEGF_121_), and abrin A chain (translation-inhibiting toxin). ABRaA**-**VEGF_121_ is strongly cytotoxic towards cells overexpressing VEGF receptor 2. The protein has antivascular properties: it destroys tumor blood vessels and triggers necrosis in adjacent areas. Both processes have inhibitory effect upon B16-F10 murine melanoma growth (Smagur et al. 2009).

Interleukin (IL-12) is a versatile cytokine capable of activating non-specific (NK and NK-T cells), as well as specific (CD4^+^, CD8^+^ cells) immune response effectors (Del Vecchio et al. 2007). IL-12 exerts influence on effector functions of T, NK and NK-T cells and induces release of interferon-γ (IFN-γ) by these cells. IFN-γ inhibits angiogenesis and remodeling of extracellular matrix (by suppressing several matrix metalloproteases activity), and stimulates release of IP-10 and MIG chemokines, which can subsequently recruit other cells that mediate non-specific and specific immune responses. IFN-γ also inhibits adhesion of endothelial cells. IL-12 effects exerted on tumors include: apoptotic death of memory T cells, numerical reduction of suppressor lymphocytes and activation of CD8^+^ effector cells (Kilinc et al. 2006; Portielje et al. 2003). IL-12 alters the character of tumor microenvironment by rendering it antiangiogenic and abolishes its immunosuppressive properties (Szala et al. 2010).

We observed that tumor vasculature-targeting therapy (mediated by ABRaA**-**VEGF_121_ protein) in combination with immunostimulatory agent (IL-12 gene therapy) inhibits tumor growth in B16-F10 murine melanoma and leads to a ca. 20 % cure of the treated mice. Decrease in tumor size was accompanied by lowered number of blood microvessels in the tumor microenvironment and lowered number of regulatory T lymphocytes, as well as by increased levels of CD4^+^ and CD8^+^. It appears that this is a promising approach to eliminate tumors.

## Materials and Methods

### Cell Culture

B16-F10 murine melanoma cells (American Type Culture Collection, Manassas, VA, USA) were grown in complete RPMI 1640 medium (Gibco BRL, Paisley, UK) supplemented with 10 % fetal bovine serum (ICN Biomedicals, Costa Mesa, CA, USA).

### In Vivo Therapy

C57Bl/6 mice (6–8 week old females) with their left dorsal side shaved were inoculated subcutaneously with B16-F10 cells (2 × 10^5^ cells per animal). On the 5th day after inoculation, when tumors reached ca. 50 mm^3^, the animals were divided into control and three therapeutic groups (*n* = 5) treated with phosphate buffered saline (PBS^−^) only, with ABRaA**-**VEGF_121_ only, with IL-12 only, or with combination of both. Chimeric recombinant protein ABRaA**-**VEGF_121_ was isolated and purified as described in Smagur et al. (2009). PBS^−^ or ABRaA**-**VEGF_121_ (0.2 mg/kg body mass) was injected intratumorally 4× every other day (on days 6, 8, 10, 12) after inoculation. Plasmid pBCMGSNeo/mIL-12 (50 μg/100 μL PBS^−^ buffer pH 7.4, Budryk et al. 2000; Mitrus et al. 2006) was administered intratumorally nine times, beginning on the 13th day after inoculation. pBCMGSNeo/mIL-12 plasmid preparations were obtained as described by Jarosz et al. (2013). Tumor volume was calculated every day starting from 5th day after inoculation, using the formula: tumor volume = (width)^2^ × length × 0.52. The experiment was repeated twice. Animals originated from own animal facility at the Center for Translational Research and Molecular Biology of Cancer. Permission for animal studies was obtained from the Local Ethics Commission (Medical University of Silesia, Katowice, Poland).

### In Vivo Post-Therapeutic Analysis

Since therapeutic experiments led sometimes to complete cure, the scheme of administering ABRaA**-**VEGF_121_ and IL-12 in the experiments intended for cytometric and histological analyses was changed. Therapeutic doses remained unchanged, but timing and frequency of their application were modified. C57Bl/6 mice were inoculated subcutaneously with B16-F10 cells and divided into four test groups (*n* = 5) as described above. ABRaA**-**VEGF_121_ was injected intratumorally twice, on 11th and 13th day. pBCMGSNeo/IL-12 was administered 4× beginning on 14th day after inoculation. Twenty-four hours following the final injection mice were sacrificed, tumor material collected and prepared for analysis. Each experiment was repeated twice.

### Histological Analysis

Tumor material was collected, formaldehyde-fixed and embedded in paraffin. Paraffin sections (5 μm) were stained either with hematoxylin and eosin (H&E), or immunohistochemically, and were observed under a light microscope. Non-specific binding was blocked by incubation in 3 % H_2_O_2_. Following incubation at 37 °C for 2 h with rabbit anti-CD31 polyclonal primary antibody (Abcam, Cambridge, UK), sections were incubated with secondary antibody labeled with horseradish peroxidase. Finally, they were incubated with DAB peroxidase substrate and, additionally, with hematoxylin.

### Flow Cytometric Analysis

Single-cell suspension was obtained using a digestion mix [0.5 mg/mL collagenase A (Sigma Aldrich, MO, USA); 0.2 mg/mL hyaluronidase type V (Sigma Aldrich, MO, USA); 0.02 mg/mL DNase I (Roche Diagnostic GmbH, Germany) per each 0.25 g of tumor tissue]. Red blood cells were lysed using 0.15 M ammonium chloride solution. Dead cells were removed by centrifugation on Lympholyte-M gradients (Cedarlane, ON, Canada). To identify the subpopulations of T lymphocytes, the following antibodies were used: PE-Cy7-CD3e, PE-CD4, FITC-CD8a (BD Bioscience, CA, USA). Treg lymphocytes were identified with FITC-CD4, APC-CD25 and PE-Foxp3 antibodies (eBiosciences, CA, USA). All flow cytometric analyses were performed using BD FACSCanto apparatus (BD, Franklin Lakes, NJ, USA). Gate parameters dividing negative from positive cells were chosen based on isotype antibody control probes (Jarosz et al. 2013).

### Statistical Analysis

Statistical comparison of tumor growth data was carried out using Mann–Whitney *U* test. Differences in the number of microvessels, levels of CD4^+^, CD8^+^, and Treg cells between the experimental groups were evaluated by analysis of variance test (ANOVA). *p* < 0.05 values were considered as statistically significant.

## Results and Discussion

Abrogation of proangiogenic and immunosuppressive character of tumor microenvironment constitutes a promising strategy to eliminate tumors (Jinushi and Dranoff 2007; Noonan et al. 2008; Szala et al. 2010; Terme et al. 2012). Inhibition of angiogenesis alters tumor milieu in a way that enhances antitumor immune response (Tartour et al. 2011). Besides abrogating immunosuppressive conditions within tumor, this strategy appears to bypass drug resistance which sets in during protracted administration of antiangiogenic agents (Ostrand-Rosenberg 2008). Thus, antiangiogenic strategy alone is not a satisfactory therapeutic approach (Azam et al. 2010). In practice, this anticancer strategy can be implemented by combining antiangiogenic and immunostimulatory drugs (Jinushi and Dranoff 2007; Noonan et al. 2008; Tartour et al. 2011). Such drug combination has been known to reduce the number of tumor microvessels and activate antitumor immune response (e.g., Jarosz et al. 2013).

Instead of combined antiangiogenic therapy, we examined antivascular therapy in combination with immunostimulatory factor. We studied whether antivascular chimeric protein ABRaA**-**VEGF_121_ and immunostimulatory cytokine IL-12 (in the form of gene therapy) inhibited growth of tumor B16-F10 murine melanoma more effectively. First, we investigated possible therapeutic effects of using the combined approach and then, following therapy, we analyzed the collected tumor tissue material. We focused on assessing the number of tumor microvessels, as well as on determining the level of particularly important immune system cells (CD4^+^, CD8^+^ and Treg) participating in the regulation of tumor microenvironment.

**Fig. 1:**
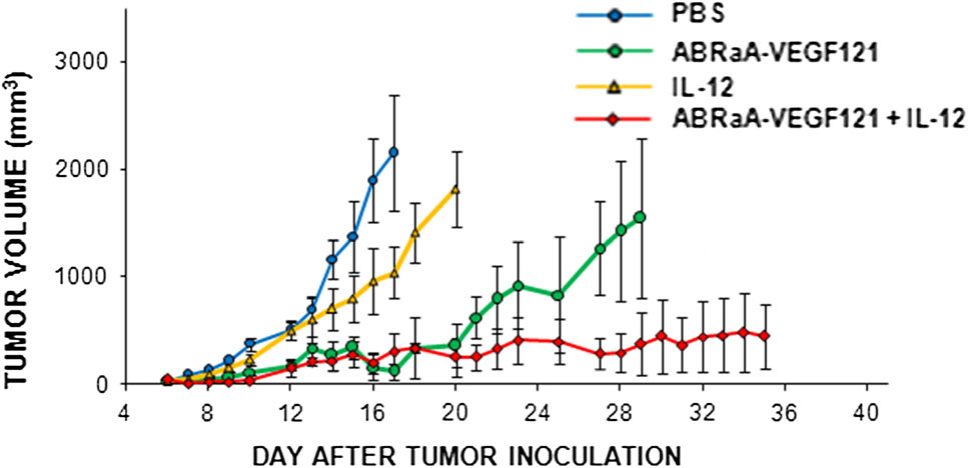
Inhibition of B16-F10 tumor growth. PBS^−^ or ABRaA**-**VEGF_121_ (0.2 mg/kg body mass) was injected intratumorally four times every other day (on days 6, 8, 10, 12) after B16-F10 inoculation. Plasmid pBCMGSNeo/mIL-12 (50 μg dose) was administered intratumorally 9× beginning on the 13th day after inoculation. Statistically significant differences (*p* < 0.05; Mann–Whitney *U* test) in tumor size among the group treated with combination therapy (ABRaA**-**VEGF_121_ + IL-12), the group treated with IL-12 and the control group were observed starting from day 10. The graph shows representative results of one of two independent experiments

Our results demonstrate that combination of ABRaA**-**VEGF_121_ antivascular agent with immunostimulatory IL-12 indeed inhibits tumor growth of B16-F10 melanoma tumors more effectively than either agent alone, leading to complete cure of ca. 20 % of the treated mice (Fig. 1). Although monotherapy with ABRaA**-**VEGF_121_ alone yielded similar growth-inhibitory effects as the investigated drug combination, cessation of protein administration caused rapid tumor relapse. In turn, monotherapy with IL-12 gave weakest inhibitory response albeit tumor relapse following conclusion of therapy was slower than in control. Differences in tumor size between mice treated with combined therapy and treated with IL-12 (as well as compared with PBS^−^ receiving control) were statistically significant from day 10 (*p* < 0.05; Mann–Whitney *U* test). In the treatment protocol used, ABRaA**-**VEGF_121_ was meant to destroy tumor vasculature and to trigger necrosis of the damaged vessels, as demonstrated by Smagur et al. (2009). In turn, administration of IL-12 was meant to prevent tumor regrowth. Administration of both therapeutic agents (ABRaA**-**VEGF_121_ first, followed by plasmid DNA encoding IL-12 gene) has shown a better profile, as compared to separate treatments; tumor growth was slowest and the effect was sustained throughout the experiment (Fig. 1).

**Fig. 2:**
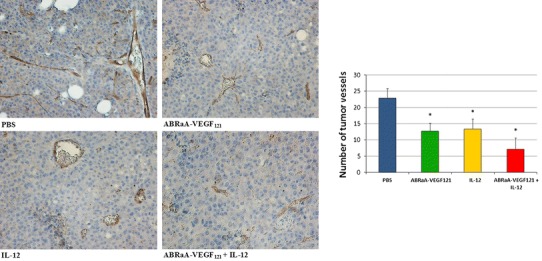
Reduction in the number of tumor blood vessels following therapy. Twenty-four hours after last intratumor injection of the drug mice (*n* = 5) were killed, tumors excised, fixed and stained with antibody against CD31. For each experimental group, the number of vessels was counted in five visual fields in four tumor sections (lens magnification ×20). The most pronounced decrease in the number of vessels was found for tumor tissue section from mice treated with combination of ABRaA**-**VEGF_121_ and IL-12. Differences in the number of vessels between control group (injected with PBS^−^) and the ones treated with IL-12, ABRaA**-**VEGF_121_ or combined therapy were statistically significant (*p < 0.05; ANOVA test). The figure shows representative results of one of two independent experiments

Post-therapeutic analysis included: comparison of tumor structure, density of tumor blood microvessels, as well as assessment of the level of tumor-infiltrating lymphocytes (CD4^+^, CD8^+^ and Treg). Since some of the animals in the therapeutic experiment were completely cured, the mice intended for post-therapeutic analysis were treated with a different scheme of administering ABRaA**-**VEGF_121_ and IL-12 (timing and frequency of their application were reduced). The greatest drop in the density of tumor blood vessels was observed in case of tumors treated with the combined regimen. Neither therapy with antivascular protein nor with IL-12 alone yielded equally good effects (Fig. 2). In addition, combined therapy brought about the greatest accretion of destroyed microvessels surrounded by areas with necrotized cancer cells. Conceivably, the necrotic debris may participate in stimulation of immune response directed against cancer cells (Zitvogel et al. 2008). Reduction of the number of tumor blood microvessels inhibits tumor growth (Niethammer et al. 2002).

**Fig. 3:**
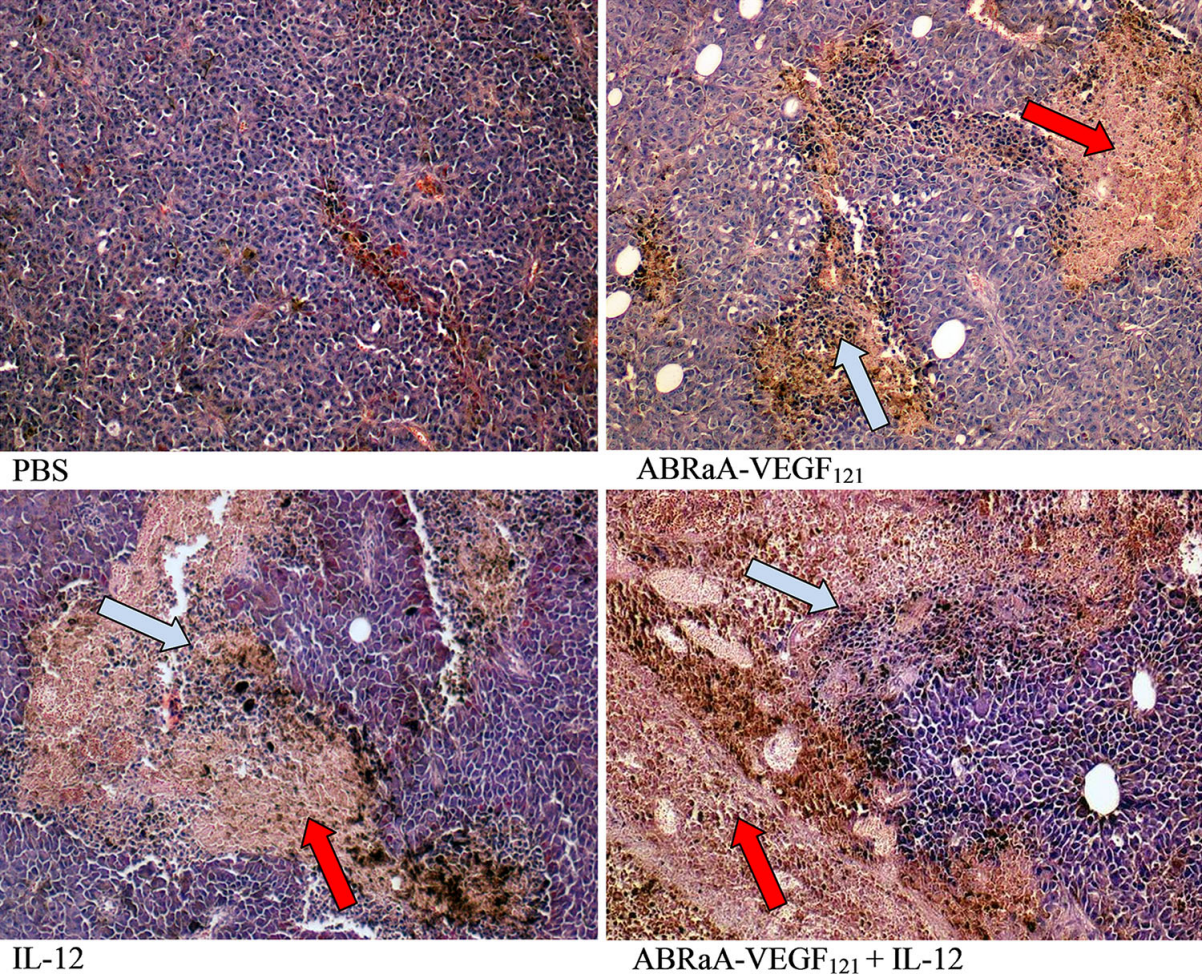
Necrotic areas and infiltration of immune cells in tumors. Mice (*n* = 5) were treated with ABRaA**-**VEGF_121_, IL-12 and combination of ABRaA**-**VEGF_121_ + IL-12. Twenty-four hours after last intratumor drug injection mice were killed, tumors excised, fixed and stained with H&E. Histological specimens demonstrate considerable necrotic areas and immune cells’ infiltration in tumor sections from all three experimental groups. Necrotic areas and infiltration were most pronounced in the group treated with combined therapy. *Red arrows* indicate necrotic areas, *blue ones* indicate immune cells infiltration. Lens magnification was ×20. The figure shows representative results of one of two independent experiments

**Fig. 4:**
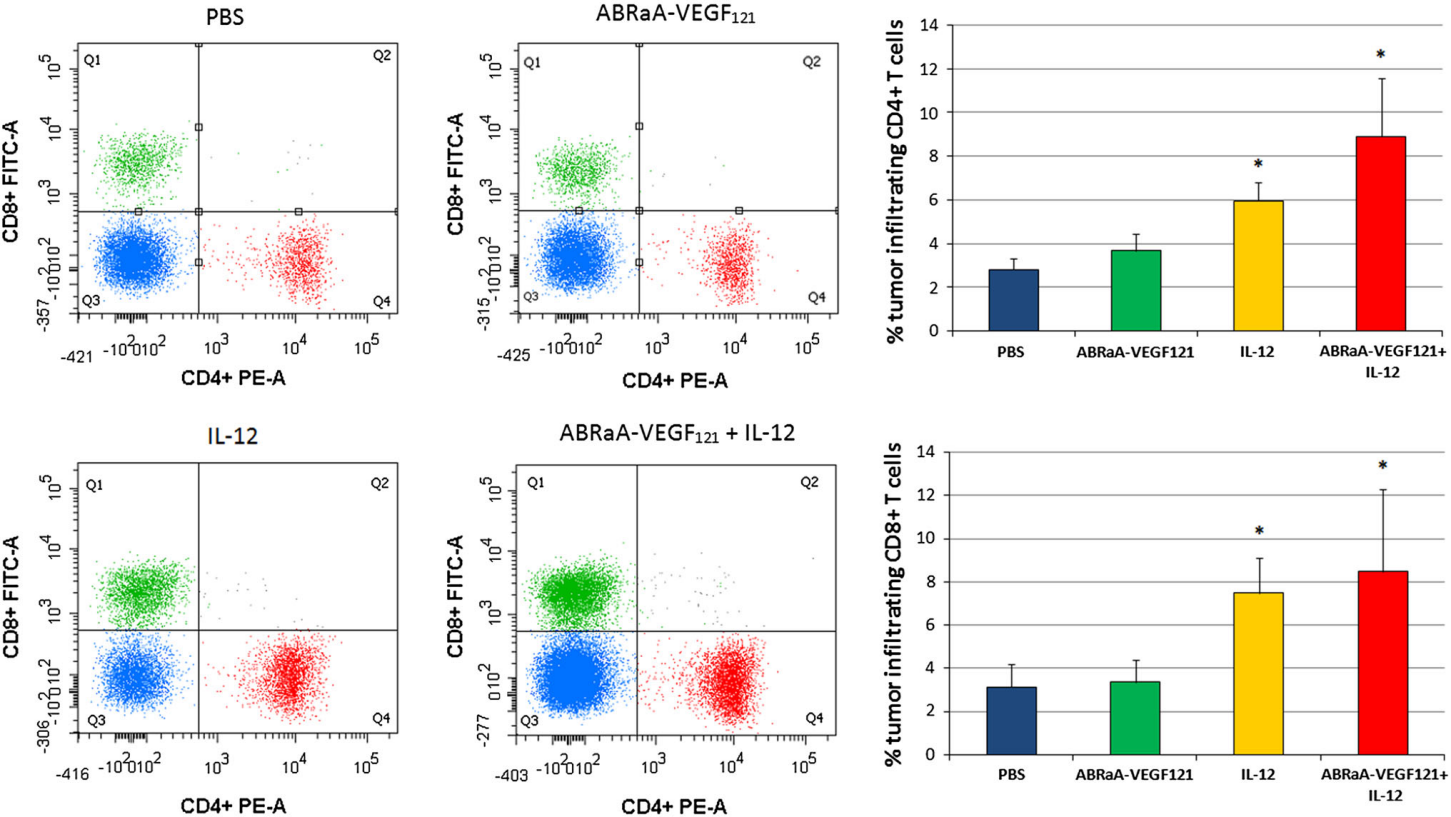
Induction of specific immune response. Twenty-four hours after last intratumor drug injection mice (*n* = 5) were killed and tumors were excised. Single-cell suspensions from B16-F10 tumors were analyzed by flow cytometry. To identify T lymphocyte subpopulations, the following antibodies were used: PE-Cy7-CD3e, PE-CD4 and FITC-CD8a. Gate dividing negative from positive cells was based on isotype antibody control probes. Significantly higher numbers of CD4^+^ and CD8^+^ were noted after therapy with IL-12, ABRaA**-**VEGF_121_ protein and after combined therapy. Differences between control group (injected with PBS^−^) and the ones treated with IL-12 or combined therapy were statistically significant (*p < 0.05; ANOVA test). The figure shows representative results of one of two independent experiments

Comparison of tumor histological features has demonstrated the presence of necrotized tissue as well as infiltration of immune cells in virtually every tumor borne by mice subjected to therapeutic intervention (Fig. 3). Immunostimulatory effect was demonstrated for all three approaches tested: ABRaA**-**VEGF_121_, IL-12 and ABRaA**-**VEGF_121_ with IL-12. The increased levels of CD4^+^ and CD8^+^ lymphocytes were slightly higher when the combined approach was used, followed by IL-12. The effect of antivascular protein was the weakest (Fig. 4). Extent of immune system cells’ infiltration was analyzed in H&E-stained tumor tissue specimens (Fig. 3).

The appearance of specific immune response cells in tumor specimens has been linked to positive therapeutic prognosis (Joyce 2005). The tumor immunoenvironment is regulated by immune cells: T lymphocytes, dendritic cells, natural killer cells, macrophages and neutrophils (Shurin et al. 2012). It has been known that CD4^+^ and CD8^+^ lymphocytes inhibit tumor development and result in the development of immune memory preventing tumor relapse (Ostrand-Rosenberg 2008). The role of CD4^+^ helper lymphocytes involves stimulation of cytotoxicity of CD8^+^ lymphocytes and activation of their proliferation and differentiation. In turn, cytotoxic CD8^+^ lymphocytes have the ability to directly destroy cancer cells by inducing their apoptosis (Castellino and Germain 2006; Ostrand-Rosenberg 2008). Increase in the number of CD4^+^ and cytotoxic CD8^+^ lymphocytes’ in tumors is inversely correlated with the level of immunosuppressive cells: Treg lymphocytes, regulatory dendritic cells and tumor-associated macrophages (Shurin et al. 2012). Accumulation of regulatory lymphocytes in tumor microenvironment shifts the balance between effector and suppressor lymphocytes and induces immunosuppressive state (Rabinovich et al. 2007; Whiteside 2006; Zou 2005). Regulatory lymphocytes inhibit proliferation of CD8^+^ lymphocytes and maturation of dendritic cells (Ostrand-Rosenberg 2008). Immature dendritic cells have been recently found to promote angiogenesis in tumors. Tumors that escape immune surveillance inhibit dendritic cell maturation (Ma et al. 2013). Fainaru et al. (2010) showed that immature dendritic cells promote angiogenesis and growth of various human and murine tumors in mice, while dendritic cell maturation inhibits this phenomenon.

**Fig. 5:**
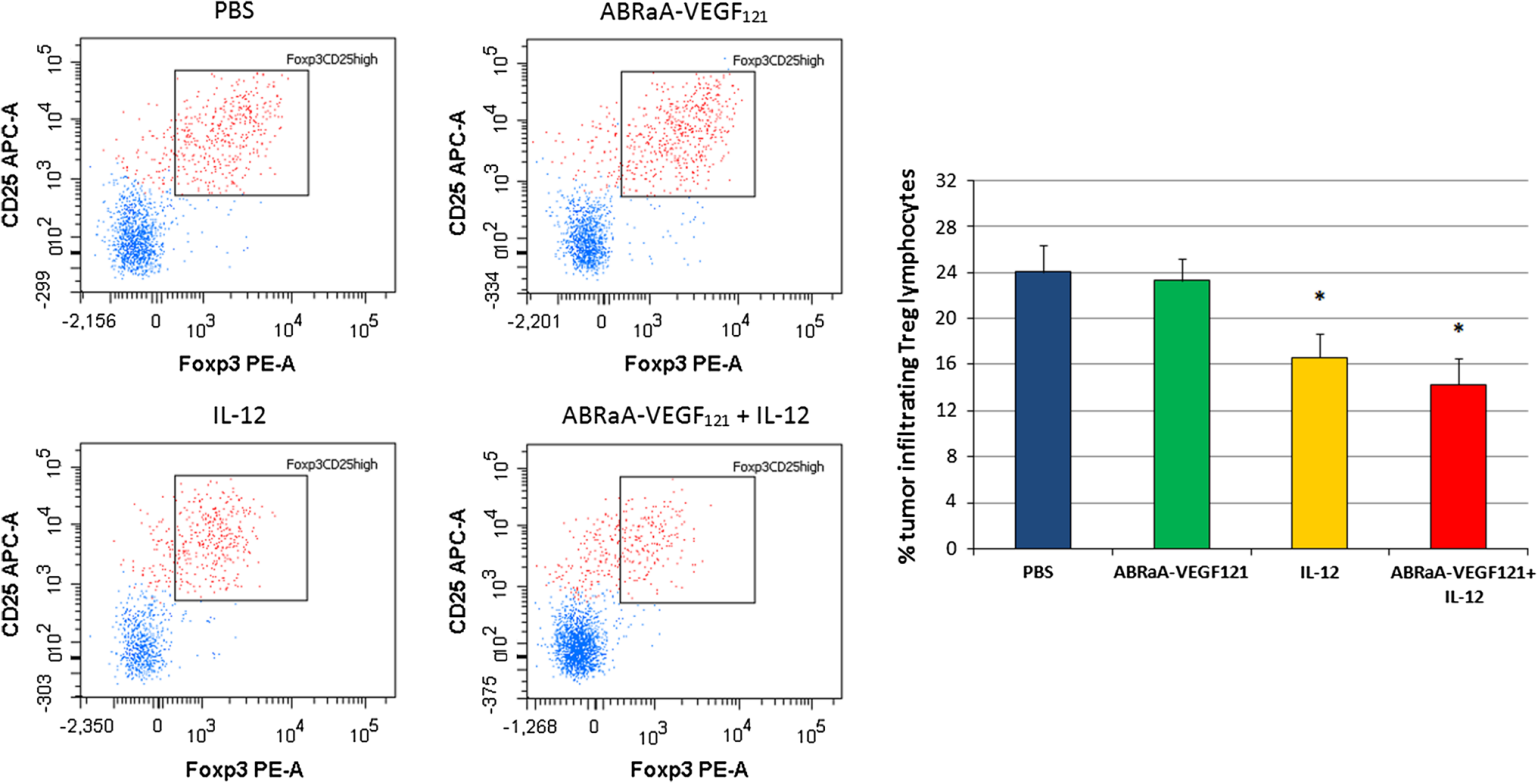
Reduced level of Treg lymphocytes following therapy. Twenty-four hours after last intratumor drug injection mice (*n* = 5) were killed and tumors were excised. Single-cell suspensions from B16-F10 tumors were analyzed by flow cytometry. Treg lymphocytes were identified using FITC-CD4, APC-CD25, and PE-Foxp3 antibodies. Percentage of Foxp3^+^CD25^+^ regulatory lymphocytes was determined from lymphocyte population gate. The largest decrease in the number of tumor Treg lymphocytes was found for the group of mice treated with combined therapy. Differences between control group (injected with PBS^−^) and the ones treated with IL-12 or combined therapy were statistically significant (*p < 0.05; ANOVA test). The figure shows representative results of one of two independent experiments

In our study, we focused on immunosuppressive Treg cells promoting tumor angiogenesis (Facciabene et al. 2012). We detected decreased levels of Treg lymphocytes in tumors treated with IL-12, as well as in tumors treated with combinatory approach (as compared to control tumors injected with PBS^−^). The effect was most visible in tumor tissue specimens treated with the combined approach. Use of ABRaA**-**VEGF_121_ protein alone did not significantly alter the level of suppressor T lymphocytes (Fig. 5). Nagai et al. (2004) showed that elimination of regulatory T cells combined with IL-12 gene transfer lead to tumor rejection of B16-F10 murine melanoma.

The results described herein indicate that the combinatory approach used (antivascular protein and immunostimulatory cytokine) indeed inhibits growth of experimental B16-F10 murine melanoma, sometimes leading to complete tumor regression. We think that the obtained effect is probably due to the polarization of tumor microenvironment. Our understanding of this polarized milieu includes such elements as: decreased number of blood microvessels, correlation with lowered levels of suppressor lymphocytes (Treg), as well as with elevated levels of CD4^+^ and CD8^+^ (Jarosz et al. 2013; Szala et al. 2010). This corroborates previous reports concerning effectiveness of a strategy combining antiangiogenic and immunostimulatory drugs (e.g., Noonan et al. 2008). IL-12 is a potent adjuvant enhancing effectiveness of antitumor therapies (Portielje et al. 2003). Our approach to therapy with IL-12 has been based on the use of a genetic construct (plasmid DNA encoding IL-12 gene), since such a modus operandi decreases the number of side effects (toxicity of therapy and strong immune reaction) (Imboden et al. 2003; Uemura et al. 2010). Polarization of tumor microenvironment has also been evident following IL-12 genetic monotherapy. This is because IL-12 exerts antiangiogenic effects and stimulates both types of immune response (Del Vecchio et al. 2007; Uemura et al. 2010). In addition, IL-12 eliminates Treg lymphocytes from tumor milieu, thus abolishing its immunosuppressive character (Jarosz et al. 2013; Kerkar and Restifo 2012). IL-12 induces IFN-γ synthesis in NK, T cells, dendritic cells, and macrophages (Hamza et al. 2010; Watford et al. 2003). It also enhances antitumor effect of a combined therapy as well as IL-12 monotherapy (Uemura et al. 2010). In turn, IL-12 affects T cells and NK cells by amplifying production and activity of cytotoxic lymphocytes and inducing proliferation and production of cytokines, especially IFN-γ (Colombo and Trinchieri 2002). IL-12 also enhances differentiation of naϊve CD4^+^ T cells into T helper 1 cells which produce IFN-γ as well as helps in cell-mediated immunity (Colombo and Trinchieri 2002; Watford et al. 2003). IFN-γ induced by IL-12 could inhibit tumor angiogenesis mediated by IFN-inducible protein 10 (Sgadari et al. 1996). IFN-γ decreased production of VEGF. IL-12 treatment reduced the production of metalloproteases which play a role in matrix remodeling during angiogenesis (Portielje et al. 2003; Uemura et al. 2010). Moreover, IL-12-induces IFN-γ, decreases activation of integrin α_v_β_3_ present on endothelial cells, leading to reduced adhesion and survival of the latter (Portielje et al. 2003; Uemura et al. 2010).

To conclude, our results indicate that the therapeutic combination involving antivascular protein ABRaA**-**VEGF_121_ and an immunostimulatory cytokine (IL-12) inhibits growth of B16-F10 murine melanoma experimental tumors and leads to total tumor cure in ca. 20 % mice. We suppose that the therapeutic effect obtained may be caused by tumor microenvironment polarization, i.e., decreased density of tumor microvasculature, lower level of suppressor T lymphocytes and increased levels of CD4^+^ and CD8^+^ cells. This allows us to conclude that bringing together antivascular strategy and the action of immunostimulating agents seems to be a promising anticancer therapeutic approach.
